# Supplementary material for: A novel CNN gap layer for growth prediction of palm tree plantlings
Source: PLoS One. 2023 Aug 11;18(8):e0289963. doi: 10.1371/journal.pone.0289963 (PMC10420369; doi:10.1371/journal.pone.0289963)
Supplement: Attachment — Submitted filename: Response to Reviewer#2.docx [file pone.0289963.s001.docx]

**Response to Reviewer 2**

**Article ID:** PONE-D-23-01139

**Original Article Title:** A NOVEL CNN GAP LAYER FOR GROWTH PREDICTION OF PALM TREE PLANTLINGS

**Re:** Response to reviewers

Dear Editor-in-chief,

Thank you for allowing a resubmission of our manuscript, with an opportunity to address the reviewers’ comments. We appreciate the Editor and the reviewers for taking the time to review our manuscript carefully and for providing valuable feedback to help us improve the quality of the document.

We have made every effort to eliminate all the indicated major and minor inconsistencies and do hope that as a result, the quality of our revised manuscript has improved further. The updated parts are highlighted.

Please see our replies to the comments of the Reviewer #2 below.

Best regards

Tarek

| **Reviewer #2** | | |
| --- | --- | --- |
| The performance of the existing CNN-based models for Counting and identifying plantlings could be further improved. To achieve this, a novel CNN architecture (CNN-SLNet) has been proposed and used for building a new IoT effective monitoring system for the prognostication of oil palm tree seedling. The proposed model is trained to predict the successful and poor seedling growth for a given set of palm tree seedling images. The proposed CNNSLNet architecture is novel in terms of defined con-volution layers and the gap layer designed for output classification. | | |
| 1 | Why the tree has the potential to reduce both the temperature of the atmosphere and the level of pollutants produced by industrial activity? You need to validate “automated monitoring and growth interpretation of palm tree seedlings gives farmers a new way to manage their resources that is based on technology instead of the old way they did it in the past”. | Thanks for your comment on this. This has been justified in the first paragraph in the introduction, **see the highlighted paragraph and it is given below too**.  “The palm tree’s health prediction system with symmetrical design has introduced a new aspect to its consequences for future environmental betterment [4]. For smart farming, the users need a reliable source of information about how things are going now. So, automated monitoring and growth interpretation of palm tree seedlings gives farmers a new way to manage their resources that is based on technology instead of the old way they did it in the past. The monitoring and growth prediction method also gives information about plant growth and health, which is especially helpful for keeping track of plants' age and death rate that will be used to grow palm trees in the future [4]. It is important to obtain reliable detection for palm trees in huge regions because of the economic gain and detrimental environmental consequences. Such precise observations would enhance plantation planning, oil palm productivity, and decrease personnel and fertilizer use by allowing for more precise monitoring of the plantations' growth and more intelligent control of the farms' operations.” |
| 2 | Why the potential to comprehend complex data is a substantial advantage of deep learning? How deep learning has been implemented to crop cultivation to cut production costs and hence increase agricultural productivity? | Conventional statistics and machine-learning approaches can have substantial issues when attempting to extract usable information from data, but deep learning mitigates this necessity [16]. One potential area of study is the use of Deep Learning algorithms for the automated extraction of high-abstraction data representations (features). These algorithms build a hierarchical structure for learning and representing data, with each layer defining the next one up in terms of the features it learned from the previous one [17].  Millions of data sets on heat, sediment, water consumption, weather condition, etc. are produced daily on farms. This data is used in real-time with the assistance of artificial intelligence and deep learning models to get insights such as the optimal time to sow seeds, crop selection, hybrid seed selection for increased yields, and so on [18]. Accurate and reliable harvesting, or "precision agriculture," has been helped along using deep learning systems. Plant diseases, pest infestations, and nutrient deficiencies can all be identified with the aid of DL technology. Using DL methods, we can pinpoint the exact location of weeds and determine the most effective herbicide to use in that specific area. By doing this, we save revenue and lessen our reliance on herbicides [19]. |
| 3 | Section 2, pls discuss related papers, fruit leaf diseases classification: a hierarchical deep learning framework, intelligent tracking of mechanically thrown objects by industrial catching robot for automated in-plant logistics 4.0, fruit category classification by fractional Fourier entropy with rotation angle vector grid and stacked sparse autoencoder. | A number of papers suggested by the reviewer are incorporated into section 2 and they are listed below.  Zhang, Yu‐Dong, Suresh Chandra Satapathy, and Shui‐Hua Wang. "Fruit category classification by fractional Fourier entropy with rotation angle vector grid and stacked sparse autoencoder." *Expert Systems* 39, no. 3 (2022): e12701.  Qadeer, Nauman, Jamal Hussain Shah, Muhammad Sharif, Muhammad Attique Khan, Ghulam Muhammad, and Yu-Dong Zhang. "Intelligent tracking of mechanically thrown objects by industrial catching robot for automated in-plant logistics 4.0." *Sensors* 22, no. 6 (2022): 2113.  Gaikwad, Sukanya S., Shivanand S. Rumma, and Mallikarjun Hangarge. "Fungi affected fruit leaf disease classification using deep CNN architecture." *International Journal of Information Technology* (2022): 1-10. |
| 4 | How recent advancements in software platform, wireless sensors, and computer vision may allow for significant time and expense reductions in plant seedling monitoring?  What is the motivation to design a novel CNN-SLNet architecture for efficient monitoring and prediction of palm tree seedling health using UAV and IoT technology? | **This has been discussed in Section 3 and given below.**  Many agronomic applications make use of wireless sensor networks, such as remote monitoring of environmental and soil conditions for estimating crop viability. Using environmental parameters such as temperature, moisture, temperature, relative humidity, salinization, and soil conductance as inputs, WSN-based applications can calculate an anticipated irrigation schedule for agricultural areas [47]. Manually detecting crop diseases and pests, using statistical calculations to forecast the amount and estimate the production and loss of crops. These were time-consuming processes and prone to human error [48]. With the use of data analytics and machine learning, we can extract the most relevant insights from agricultural data and improve precision in farming. Vector models, neural network, regression techniques, fuzzy logic, RNN and CNN are some of the most popular and relevant machine learning techniques used in weather-based crop monitoring [49].  Digital mounted cameras on lightweight unmanned aircraft systems can capture images of the plantlings. Object-detection algorithms like CNN can then be applied to the acquired imagery to assess the growth of plant plantlings. But still the attempt of palm tree seedling monitoring is not yet done in any research. Hence in this study, we design a novel GL-CNN architecture for efficient monitoring and prediction of palm tree seedling health with UAV for image collection and IoT technology for ground truth estimation. |
| 5 | Why this makes it challenging to keep a close eye on the seeds and young plants. Restoration professionals require technical approaches that can provide them with high resolution, rapid, and scalable plant-based monitoring systems? | Conventional approaches to plantlings' health tracking have made use of remote sensing tools like proximity sensing devices, spectrometry, machine vision systems, mapping techniques, and drones. There are still some partial difficulties with these technologies that prevent them from being widely used for tailored and long-term plantlings monitoring [17]. These methods are not suitable for precise monitoring of plant growth, or the prolonged tracking of the plant’s growth. They do not have high spatial or temporal resolution and they can only perform discontinued measurements. In the past, tracking seedlings required conducting costly and time-consuming field surveys during which seedlings had to be located, named, and counted while still in the soil [10]. Machine learning and deep learning architectures are the newest technologies that improve ecological monitoring. |
| 6 | Description of CNN is unclear. You can refer to two-stage intelligent darknet-squeezenet architecture-based framework for multiclass rice grain variety identification, a five-layer deep convolutional neural network with stochastic pooling for chest CT-based covid-19 diagnosis. | The description of CNN is rephrased, and additional details are added up. Moreover a new architectural diagram is presented. |
| 7 | Why Baye’s optimizing tests also confirmed that ReLU and dropout have collaboration, which implies that using them together is optimal? | Thanks for your comment on this.  To reduce dropout and overfitting problems on a global scale, Bayesian optimization (BO), the statistical optimization method, was used. BO is used to minimize or maximize an objective function to guide the search. When implemented for hyperparameter tuning in our method, the ReLU objective function becomes costly to evaluate. This can be eradicated by realizing the Bayes technique for parameter tuning in ReLU. |
| 8 | Virtue of global average polling is not clear. Please check “Global average pooling has virtue over fully connected layers (FC) layers in that it promotes correspondences amongst feature maps and subcategories, making it more ideal for the convolution layout. As a corollary, the extracted features may be simply under-stood as category optimism maps” | **This is a good point, thanks for making it.**  Global average pooling aggregates geometric information, making it even more susceptible to source spatial changes. In conventional CNNs, fully connected layers are typically replaced with a pooling technique called global average pooling. The idea of the final conv layer is to build a feature map for each class that has been established through the classification process. To avoid the need for additional fully linked layers on top of the feature maps, we simply average the maps and feed the resulting vector into the softmax layer. |
